# White blood cell count and incidence of hypertension in the general Japanese population: ISSA-CKD study

**DOI:** 10.1371/journal.pone.0246304

**Published:** 2021-02-02

**Authors:** Shintaro Ishida, Seiji Kondo, Shunsuke Funakoshi, Atsushi Satoh, Toshiki Maeda, Miki Kawazoe, Chikara Yoshimura, Kazuhiro Tada, Koji Takahashi, Kenji Ito, Tetsuhiko Yasuno, Kosuke Masutani, Hitoshi Nakashima, Hisatomi Arima

**Affiliations:** 1 Department of Oral and Maxillofacial Surgery, Faculty of Medicine, Fukuoka University, Fukuoka, Japan; 2 Department of Preventive Medicine and Public Health, Faculty of Medicine, Fukuoka University, Fukuoka, Japan; 3 Division of Nephrology and Rheumatology, Department of Internal Medicine, Faculty of Medicine, Fukuoka University, Fukuoka, Japan; International University of Health and Welfare, School of Medicine, JAPAN

## Abstract

**Objectives:**

This study aimed to clarify the relationship between the white blood cell (WBC) count and hypertension in the general Japanese population.

**Methods:**

We conducted a population-based retrospective cohort study using annual health check-up data of residents of Iki City, Nagasaki Prefecture, Japan. A total of 2935 participants without hypertension at baseline were included in the present analysis. WBC counts were classified as tertile 1 (<4700/μL), tertile 2 (4700–5999/μL), and tertile 3 (≥6000/μL). The outcome was incident hypertension (blood pressure ≥140 mmHg). Multivariable-adjusted hazard ratios and 95% confidence intervals (95% CIs) were estimated using the Cox proportional hazards model.

**Result:**

During an average follow-up of 4.5 years, 908 participants developed hypertension. The incidence (per 100 person-years) of hypertension increased with an elevation in the WBC count (6.3 in tertile 1, 7.0 in tertile 2, and 7.4 in tertile 3). This association was significant, even after adjustment for other risk factors, including age, sex, current smoking habits, current alcohol intake, exercise habits, obesity, elevated blood pressure, diabetes mellitus, and dyslipidemia. The hazard ratios were 1.07 for tertile 2 (95% CI 0.90–1.26) and 1.27 for tertile 3 (95% CI 1.06–1.51) compared with the reference group of tertile 1 (p = 0.009).

**Conclusion:**

The WBC count was associated with future development of hypertension in the general Japanese population.

## Introduction

Cardiovascular disease is a leading cause of premature death in Japan, as well as in other countries in the world [1, 2]. Approximately 50% of deaths from cerebrovascular disease and 59% from coronary artery disease are attributable to hypertension [2, 3]. In Japan, the average blood pressure (BP) value of the population has decreased, which is mainly due to an increase in the number of people who have received BP-lowering medication and improved management [2–5]. However, the prevalence of hypertension has not decreased during the past few decades [2–5]. Effective prevention of hypertension and subsequent cardiovascular disease requires strategies that are based on current knowledge of risk factors in Japan.

Chronic inflammation is a risk factor of hypertension [1, 2, 4–9]. However current evidence of this risk factor is mainly based on the association between high-sensitivity C-reactive protein and hypertension, and is mainly derived from Western populations [10–13]. Therefore, whether the white blood cell (WBC) count can also predict development of hypertension in the Asian population is unclear [14]. Therefore, this study aimed to clarify the relationship between the WBC count and hypertension in the general Japanese population.

## Subjects and methods

### Study design and participants

We used data from the Iki City Epidemiological Study of Atherosclerosis And Chronic Kidney Disease (ISSA-CKD), which is a population-based retrospective cohort study of the residents of Iki City, Nagasaki Prefecture, Japan. Details of the ISSA-CKD study have been described previously [15]. Iki City consists of a group of islands that are located in the north of Nagasaki Prefecture. The total population of Iki City is approximately 27,000.

Between 2008 and 2017, a total of 7895 residents aged 30 years or older underwent annual health checks that were conducted by the local government of Iki City. We excluded 1881 residents with a follow-up duration of <1 year, 2812 who had hypertension (BP ≥140/90 mm Hg or use of BP-lowering medications) at baseline, and 267 with missing information on the WBC count at baseline. Finally, a total of 2935 participants were included in the present analysis. This study was approved by the Fukuoka University Clinical Research and Ethics Centre (No. 2017M010).

### Data collection

At each health check-up, height and weight were measured with the participant wearing light clothes without shoes, and body mass index (BMI, kg/m^2^) was calculated. Obesity was defined as a BMI ≥25 kg/m^2^ [16]. BP was measured by trained staff in the right upper arm using mercury, automated, or aneroid sphygmomanometers with appropriately-sized cuffs, after at least 5 min of rest in a sitting position, in accordance with standardized guidelines [17]. BP was measured twice and the mean of the two values was used in the present analysis. Elevated BP was defined as a BP level of 130–139/80–89 mmHg [2].

Casual blood and urine samples were collected. The WBC count was determined using a multi-item automatic hemocytometer (XN-1000®; Sysmex Corporation, Kobe, Japan). Participants were classified into tertile groups of the WBC count as follows: tertile 1 (<4700/μL), tertile 2 (4700–5999/μL), and tertile 3 (≥6000/μL). Plasma glucose concentrations were determined using an enzymatic method and glycated hemoglobin (HbA1c) levels (NGSP value) were determined using high-performance liquid chromatography. The presence of diabetes was defined by a fasting glucose concentration ≥7.0 mmol/L, a non-fasting glucose concentration ≥11.1 mmol/L, HbA1c value ≥6.5% [18], or the use of glucose-lowering therapies. Serum low-density lipoprotein (LDL) cholesterol, high-density lipoprotein (HDL) cholesterol, and triglyceride concentrations were determined enzymatically. Dyslipidemia was defined by LDL cholesterol concentrations ≥3.62 mmol/l, HDL cholesterol concentrations <1.03 mmol/l, triglyceride concentrations ≥1.69 mmol/l [2], or the use of lipid-lowering medication. Information regarding the participants’ smoking habits, alcohol intake, and regular exercise was obtained using a standard questionnaire. Current smokers were defined as participants who had smoked 100 cigarettes or more, or those who had smoked regularly for more than 6 months at baseline. Alcohol intake was classified into current daily drinking or no daily drinking. Regular exercise was defined as exercise habits of ≥30 minutes per day, two times or more per week.

### Definition of outcome

The outcome of the present analysis was development of hypertension (BP ≥140 mmHg or initiation of BP-lowering medications), which was confirmed at the end of follow-up.

### Statistical analysis

Continuous variables are expressed as the mean ± SD, and trends across tertile groups of the WBC count were tested using simple regression models. Categorical variables are expressed as the number of participants (percentage), and trends across groups were tested using logistic regression models. Incidence rates of hypertension were calculated using the person-year approach. Crude and multivariable-adjusted hazard ratios (HRs) and their 95% confidence intervals (95% CIs) were estimated using the Cox proportional hazards model. The effects of the WBC count on development of hypertension were compared between subgroups defined by other risk factors (age, sex, smoking, obesity, and dyslipidemia) by adding an interaction term to the statistical models. A two-tailed p value of <0.05 was considered statistically significant. All data analyses were carried out using SAS version 9.4.

## Results

Table 1 shows the baseline characteristics according to tertile groups of the WBC count. Participants with a higher WBC count were younger and more likely to be men, with higher rates of current smoking, current daily alcohol intake, obesity, and dyslipidemia.

**Table 1:** Baseline characteristics according to tertiles of the white blood cell count.

	White blood cell count (/μL)			p value for trend
	<4,700/μL (N = 951)	4,700–5,999/μL (N = 1040)	≥6,000/μL (N = 944)	
Age, mean (SD), years	59.0 (10.7)	57.5 (11.4)	54.3 (12.1)	<0.0001
Male, N (%)	273 (28.7)	472 (45.4)	505 (53.5)	<0.0001
Smoking, N (%)	81 (8.5)	157 (15.1)	361 (38.2)	<0.0001
Current daily alcohol intake, N (%)	127 (13.5)	218 (21.2)	230 (24.7)	<0.0001
Regular exercise*, N (%)	273 (25.5)	252 (24.6)	214 (23.1)	0.2290
Body mass index, mean (SD), kg/m^2^	22.4 (3.1)	23.0 (3.2)	23.5 (3.3)	<0.0001
Obesity**, N(%)	168 (17.7)	250 (24.0)	274 (29.0)	<0.0001
Systolic blood pressure, mean (SD), mmHg	118.4 (12.1)	119.2 (12.1)	118.8 (11.9)	0.4983
Diastolic blood pressure, mesa (SD), mmHg	69.7 (8.6)	70.9 (8.2)	70.8 (8.8)	0.0046
Elevated blood pressure***, N (%)	292 (30.7)	368 (35.4)	303 (32.1)	0.5154
Diabetes mellitus****, N (%)	37 (3.8)	53 (5.1)	55 (5.8)	0.0525
HbA1c, mean (SD)	5.1 (0.5)	5.1 (0.7)	5.2 (0.8)	0.0128
High-density lipoprotein cholesterol, mean (SD), mmol/L	1.70 (0.42)	1.63 (0.44)	1.55 (0.40)	<0.0001
Low-density lipoprotein cholesterol, mean (SD), mmol/L	3.14 (0.80)	3.19 (0.82)	3.23 (0.84)	0.0251
Triglyceride, mean (SD), mmol/L	1.08 (0.76)	1.25 (0.31)	1.46 (1.12)	<0.0001
Dyslipidemia*****, mean (SD), mmol/L	332 (34.1)	250 (24.0)	274 (29.0)	<0.0001

*Exercise habits of ≥30 minutes per day for twice or more per week.

** Body mass index of ≥25 kg/m^2^.

*** BP of 130–139/80–89 mmHg. Body mass index of ≥25 kg/m^2^.

****Casual serum glucose concentrations ≥11.1 mmol/L, HbA1c values ≥6.5%, or use of glucose-lowering therapy.

*****LDL cholesterol concentrations ≥3.62 mmol/L, HDL cholesterol concentrations <1.03 mmol/L, triglyceride concentrations ≥1.69 mmol/L, or use of lipid-lowering medication.

During an average follow-up of 4.5 years, 908 participants developed hypertension. Table 2 shows the risks of hypertension according to tertile groups of the WBC count. The incidence rate of hypertension increased with elevation of the WBC count (6.3% per 100 person-years in tertile 1, 7.0% in tertile 2, and 7.4% in tertile 3). This association was significant after adjustment for other risk factors, including age, sex, current smoking habits, current alcohol intake, exercise habits, obesity, elevated BP, diabetes, and dyslipidemia (HRs of 1.07 [95% CI 0.90–1.26] for tertile 2 and 1.27 [1.06–1.51] for tertile 3 compared with tertile1 (p = 0.009).

**Table 2:** Risk of hypertension according to tertiles of the white blood cell count.

	White blood cell count			
	<4,700/μL (N = 951)	4,700–5,999/μL (N = 1040)	≥6,000/μL (N = 944)	p value for trend
N of events / person-years	273/4353	332/4736	303/4097	
Annual incidence rate	6.3%	7.0%	7.4%	
Crude hazard ratio (95% confidence interval)	1.00 (Reference)	1.12 (0.95–1.31)	1.17 (1.00–1.38)	0.056
Adjusted hazard ratio* (95% confidence interval)	1.00 (Reference)	1.07 (0.90–1.26)	1.27 (1.06–1.51)	0.009

*Adjusted for age, sex, smoking, current daily alcohol intake, exercise, obesity, elevated blood pressure, diabetes mellitus, and dyslipidemia.

Table 3 shows multivariable-adjusted HRs of the WBC count for the incidence of hypertension in subgroups. There were no clear differences in the effects of the WBC count on hypertension in subgroups defined by age (<65 vs. ≥65 years), sex, obesity, smoking, and dyslipidemia (all p>0.1 for interactions).

**Table 3:** Multivariable-adjusted hazard ratios of the white blood cell count for the incidence of hypertension in subgroups.

	White blood cell count			p value for interaction
	<4,700/μL (N = 951)	4,700–5,999/μL (N = 1040)	≥6,000/μL (N = 944)	
Age				
<65 years	1 (reference)	1.09 (0.88–1.36)	1.20 (0.96–1.51)	0.109
≥65 years	1 (reference)	1.01 (0.78–1.30)	1.08 (0.81–1.44)	
Gender				
Male	1 (reference)	1.09 (0.83–1.43)	1.07 (0.80–1.42)	0.775
Female	1 (reference)	0.98 (0.79–1.21)	1.10 (0.87–1.38)	
Current smoking				
Yes	1 (reference)	0.74 (0.44–1.24)	0.86 (0.54–1.38)	0.371
No	1 (reference)	1.09 (0.92–1.31)	1.34 (1.10–1.62)	
Obesity				
Yes	1 (reference)	1.28 (0.93–1.75)	1.34 (0.96–1.86)	0.577
No	1 (reference)	0.99 (0.81–1.20)	1.22 (0.99–1.52)	
Dyslipidemia				
Yes	1 (reference)	1.00 (0.78–1.29)	1.21 (0.93–1.57)	0.889
No	1 (reference)	1.12 (0.90–1.40)	1.29 (1.01–1.65)	

Values are hazard ratios (95% confidence intervals) adjusted for age (except for subgroup analysis by age), sex (except for subgroup analysis by sex), current smoking (except for subgroup analysis by current smoking), current alcohol intake, regular exercise, obesity (except for subgroup analysis by obesity), elevated blood pressure, and diabetes and dyslipidemia (except for subgroup analysis by dyslipidemia). Obesity: body mass index ≥25 kg/m^2^. Dyslipidemia: LDL cholesterol concentrations ≥3.62 mmol/l, HDL cholesterol concentrations <1.03 mmol/l, triglyceride concentrations ≥1.69 mmol/l, or use of lipid-lowering medications.

## Discussion

The present observational study of the general Japanese population showed a close association between the WBC count and future development of hypertension. This association was significant after adjustment for the effects of confounding factors, such as age, sex, current smoking habits, current alcohol intake, regular exercise habits, obesity, elevated BP, diabetes, and dyslipidemia. There were also similar associations between the WBC count and hypertension across subgroups defined by age, sex, smoking, obesity, and dyslipidemia.

A number of previous observational studies reported associations between indicators of inflammation, such as C-reactive protein, interleukin-6, interleukin-8, WBC count, neutrophil count, neutrophil to lymphocyte ratio, and incidence of hypertension [10–13, 19–21]. A prospective cohort study of atomic bomb survivors in Japan reported that an elevation in the WBC and neutrophil counts, which were measured in the 1960’s, were clearly related to an increased risk of future development of hypertension [22]. Our study supports findings from previous studies and showed that there was a significant association between the WBC count and the incidence of hypertension in the Japanese general population in the current era.

The mechanisms underlying the relationship between WBC count and hypertension has not been clearly defined. An increase in the number of WBCs in blood may promote leukocyte–endothelial cell adhesion, which plays a major role in development and progression of atherosclerosis [14, 19, 23, 24]. Therefore, long-term exposure to increased numbers of WBCs in blood may result in an increase in arterial stiffness and subsequent development of systemic hypertension. Another possibility is that WBC count may in part be associated with hypertension in part as a maker of obesity, because obesity is associated with higher WBC count [25] and weight gain [26] has been shown to be associated with increasing levels in blood pressure levels.

Although this was a large-scale study of the general Japanese population, it has some limitations. First, because of the retrospective nature of the study design, the findings of the present analysis may have been affected by selection bias. Second, people who were aware of healthy behavior were more likely to have attended the health check-ups and to have been included in the present analysis than those with an unhealthy lifestyle. Third, because frequency of people aged 65 years or older (“aging rate”) was higher in the Iki City (35.5% in 2015) than that of the total Japan (26.5%in 2015), our findings may not be applicable to other regions of Japan with lower aging rates. Fourth, the actual data of the new onset of hypertension was uncertain because some participants did not return to follow up to health check-up regularly. Fifth, although some studies have reported the relationship of the neutrophil count or neutrophil to lymphocyte ratio with hypertension, we do not have information of the WBC fraction.

## Conclusion

In conclusion, WBC count was associated with future development of hypertension in the general Japanese population. A high-risk strategy under guidance of the WBC count may provide better prevention of future development of hypertension.

## Supporting information

S1 Fig Hypertension incidence by tertile of WBC count. (DOCX)

S2 Fig Risk of developing hypertension by tertile of WBC count. (DOCX)

## References

[1] James SL, Abate D, Abate KH, Abay SM, Abbafati C, Abbasi N, et al Global, regional, and national incidence, prevalence, and years lived with disability for 354 diseases and injuries for 195 countries and territories, 1990–2017: a systematic analysis for the Global Burden of Disease Study 2017. Lancet. 2018; 392:1789–1858. 10.1016/S0140-6736(18)32279-7 30496104 PMC6227754

[2] Umemura S, Arima H, Arima S, Asayama K, Dohi Y, Hirooka Y, et al The Japanese Society of Hypertension Guidelines for the Management of Hypertension (JSH 2019). Hypertens Res. 2019; 42:1235–1481. 10.1038/s41440-019-0284-9 31375757

[3] Kinoshita M, Yokote K, Arai H, Iida M, Ishigaki Y, Ishibashi S, et al Japan Atherosclerosis Society (JAS) Guidelines for Prevention of Atherosclerotic Cardiovascular Diseases 2017. J Atheroscler Thromb. 2018; 2:864–984. 10.5551/jat.GL2017 30135334 PMC6143773

[4] Satoh A, Arima H, Ohkubo T, Nishi N, Okuda N, Ae R, et al Associations of socioeconomic status with prevalence, awareness, treatment, and control of hypertension in a general Japanese population: NIPPON DATA2010. J Hypertens. 2017; 35:401–408. 10.1097/HJH.0000000000001169 28005709

[5] Fujiyoshi A, Ohkubo T, Miura K, Murakami Y, Nagasawa S-y, Okamura T, et al Blood pressure categories and long-term risk of cardiovascular disease according to age group in Japanese men and women. Hypertens Res. 2012;35:947–953. 10.1038/hr.2012.87 22739419

[6] Martin D, Wallace D, Crowe M, Rush C, Tosenovsky P, Golledge J. Association of total white cell count with mortality and major adverse events in patients with peripheral arterial disease: a systematic review. Eur J Vasc Endovasc Surg. 2014; 47:422–432. 10.1016/j.ejvs.2013.12.020 24485842

[7] Whelton WP, Carey RM, Aronow WS, Casey DE Jr, Collin KJ, Himmelfarb CD, et al 2017 Guideline for the Prevention, Detection, Evaluation, and Management of High Blood Pressure in Adults. J Am Coll Cardiol; 2017. 10.1016/j.jacc.2017.11.006 29146535

[8] Kim DJ, Noh JH, Lee BW, Choi YH, Chung JH, Min YK, et al The associations of total and differential white blood cell counts with obesity, hypertension, dyslipidemia and glucose intolerance in a Korean population. J Korean Med Sci. 2008; 23:193–198. 10.3346/jkms.2008.23.2.193 18436999 PMC2526447

[9] Jung CH, Lee WY, Kim BY, Park SE, Rhee EJ, Park CY, et al The risk of metabolic syndrome according to the white blood cell count in apparently healthy Korean adults. Yonsei Med J. 2013; 54:615–620. 10.3349/ymj.2013.54.3.615 23549805 PMC3635622

[10] Nakanishi N, Sato M, Shirai K, Suzuki K, Tatara K. White blood cell count as a risk factor for hypertension; a study of Japanese male office workers. J Hypertens. 2002; 20:851–857. 10.1097/00004872-200205000-00018 12011644

[11] Lakoski SG, Cushman M, Palmas W, Blumenthal R, D’Agostino RB, Herrington DM. The Relationship Between Blood Pressure and C-Reactive Protein in the Multi-Ethnic Study of Atherosclerosis (MESA). J Am Coll Cardiol. 2005; 46:1869–1874. 10.1016/j.jacc.2005.07.050 16286174

[12] Lakoski SG, Cushman M, Siscovick DS, Blumenthal RS, Palmas W, Burke G, et al The relationship between inflammation, obesity and risk for hypertension in the Multi-Ethnic Study of Atherosclerosis (MESA). J Hum Hypertens. 2011; 25:73–79. 10.1038/jhh.2010.91 20944659 PMC4066617

[13] Ebong IA, Schreiner P, Lewis CE, Appiah D, Ghelani A, Wellons M. The association between high-sensitivity C-reactive protein and hypertension in women of the CARDIA study. Menopause. 2016; 23:662–668. 10.1097/GME.0000000000000609 27093616 PMC4874866

[14] Jayedi A, Rahimi K, Bautista LE, Nazarzadeh M, Zargar MS, Shab-Bidar S. Inflammation markers and risk of developing hypertension: a meta-analysis of cohort studies. Heart. 2019; 105: 686–692. 10.1136/heartjnl-2018-314216 30700522 PMC6588169

[15] Maeda T, Yoshimura C, Takahashi K, Ito K, Yasuno T, Abe Y, et al Usefulness of the blood pressure classification in the new 2017 ACC/AHA hypertension guidelines for the prediction of new-onset chronic kidney disease. J Hum Hypertens. 2019 10.1038/s41371-019-0198-7 31113986

[16] The examination committee of criteria for ‘Obesity Disease’ in Japan JSftSoO. New criteria for ‘obesity disease’ in Japan. Circ J. 2002; 66:987–992. 10.1253/circj.66.987 12419927

[17] The Japanese Society of Cardiovascular Disease Prevention. Handbook for cardiovascular prevention. Hokendojinsha; 2014.

[18] The Japan Diabetes Society. Management of diabetes 2018-2019.Bunkodo; 2018.

[19] Wang H, Hu Y, Geng Y, Wu H, Chu Y, Liu R, et al The relationship between neutrophil to lymphocyte ratio and artery stiffness in subtypes of hypertension. J Clin Hypertens. 2017; 19:780–785. 10.1111/jch.13002 28480636 PMC8031274

[20] Belen E, Sungur A, Sungur MA, Erdoğan G. Increased Neutrophil to Lymphocyte Ratio in Patients With Resistant Hypertension. J Clin Hypertens. 2015; 17:532–537. 10.1111/jch.12533 25807989 PMC8031939

[21] Siedlinski M, Jozefczuk E, Xu X, Teumer A, Evangelou E, Svhnabel RB. White Blood Cells and Blood Pressure.A Mendelian Randomization Study. Circulation. 2020; 141:1307–1317. 10.1161/CIRCULATIONAHA.119.045102 32148083 PMC7176352

[22] Tatsukawa Y, Hsu WL, Yamada M, Cologne JB, Suzuki G, Yamamoto H, et al White blood cell count, especially neutrophil count, as a predictor of hypertension in a Japanese population. Hypertens Res. 2008; 31:1391–1397. 10.1291/hypres.31.1391 18957810

[23] Agita A, Alsagaff MT. Inflammation, immunity, and hypertension. Acta Med Indones 2017;49:158–165. 28790231

[24] Dinh QN, Drummond GR, Sobey CG, Chrissobolis S. Roles of inflammation, oxidative stress, and vascular dysfunction in hypertension. Biomed Res Int. 2014;2014:406960 10.1155/2014/406960 25136585 PMC4124649

[25] Furuncuoğlu Y, Tulgar S, Dogan AN, Calar S, Tulgar YK, Cakiroglu B. How obesity affects the neutrophil/lymphocyte and platelet/lymphocyte ratio. systemic immune-inflammatory index and platelet indices: a retrospective study. Eur Rev Med Pharmacol Sci. 2016;20:1300–1306. 27097950

[26] Fantin F, Giani A, Zoico E, Rossi AP, Mazzali G, Zamboni M. Weight Loss and Hypertension in Obese Subjects. Nutrients. 2019;11:1667 10.3390/nu11071667 31330870 PMC6682923

