# Supplementary material for: White blood cell count and incidence of hypertension in the general Japanese population: ISSA-CKD study
Source: PLoS One. 2021 Feb 2;16(2):e0246304. doi: 10.1371/journal.pone.0246304 (PMC7853436; doi:10.1371/journal.pone.0246304)
Supplement: S1 Fig — (DOCX) [file pone.0246304.s001.docx]

S1. Hypertension incidence by tertile of WBC count
