# Supplementary material for: White blood cell count and incidence of hypertension in the general Japanese population: ISSA-CKD study
Source: PLoS One. 2021 Feb 2;16(2):e0246304. doi: 10.1371/journal.pone.0246304 (PMC7853436; doi:10.1371/journal.pone.0246304)
Supplement: Attachment — Submitted filename: Response to Reviewers 2.docx [file pone.0246304.s003.docx]

**Response to Journal Requirements**

Thank you for your useful suggestions. Requests 1 and 2 presented have been corrected in Revised Manuscript with Track Changes.

**Response to Reviewer #1**

Thank you for your useful suggestions. We have attempted to address your suggestions as follows:

**Major comment 1.** Although it is a retrospective analysis, it is an interesting study that analyzes the association between the development of hypertension and white blood cell (WBC) count in many subjects. However, more detail discussion should be given to the causal relationship between WBC count and hypertension. For example, if it is reported that the number of WBC decreases due to improvement of obesity and lipid abnormalities, it becomes easier to support the results of this study.

**Response.** Thank you for your advice. (1) We could not find a paper that reported that WBC decreased due to improvement of obesity but there was a cross sectional study that reported the association between obesity and higher WBC counts. Because weight gain has been shown to be associated with increase in blood pressure levels, WBC counts may in part be associated with hypertension in part as a maker of obesity. We have added this the 3^rd^ paragraph of the Discussion section (lines 167 to 170, page 8) as follows:

“Another possibility is that WBC count may in part be associated with hypertension in part as a maker of obesity, because obesity is associated with higher WBC count [25] and weight gain [26] has been shown to be associated with increasing levels in blood pressure levels. “

(2) We could find papers which reported that WBC decreased due to lipid lowering treatment using statins. However, because statins have anti-inflammatory effects, it is not clear whether lowering lipid abnormalities lowering itself reduces WBC counts or not. Therefore, we are not sure whether lipids are involved in the link between WBC counts and hypertension.

**Major comment 2.** It is necessary to correct LDH cholesterol to LDL cholesterol in Table 2.

**Response.** Thank you for your advice. We suppose that the typo you pointed out is in Table1 instead of Table2. We corrected “LDH cholesterol” to “LDL cholesterol” in the revised Table1.

**Response to Reviewer #2**

Thank you for your useful suggestions. We have attempted to address your suggestions as follows:

**Major comment 1.** Iki-City is considered to be an aging society compared to the national average in Japan. It may affect as selection bias.

**Response.** As you pointed out, frequency of people aged 65 years or older (“aging rate”) was 35.5% in the Iki City in 2015, which was higher than that of the total Japan (26.5%in 2015). As a result, findings of this study with higher frequency of elderly may not be applicable to other regions of Japan with lower aging rates. We have added this limitation to the 4^th^ paragraph of the Discussion section (lines 176 to 178, page 8) as follows:

“Third, because frequency of people aged 65 years or older (“aging rate”) was higher in the Iki City (35.5% in 2015) than that of the total Japan (26.5%in 2015), our findings may not be applicable to other regions of Japan with lower aging rates.”

**Major comment 2.** A number of previous observational studies reported associated between indicators of inflammation and incidence of hypertension. A prospective cohort study of a Japanese population was also reported. What is the novelty in the present study?

**Response.** Thank you for your advice. A novelty of the present study is the fact that this is a population-based study in current Japan. Although one longitudinal study investigated the association between white blood cell counts and incidence of hypertension among Japanese, their participants were atomic bomb survivors in the Hiroshima and the Nagasaki Prefectures. Furthermore, baseline assessment of white blood cells counts were conducted in 1960’s. In contrast, participants of this study were general Japanese people and baseline assessment was conducted after 2006.

Yours sincerely

Shintaro Ishida
